# Transcriptomic profiling of tall fescue in response to heat stress and improved thermotolerance by melatonin and 24-epibrassinolide

**DOI:** 10.1186/s12864-018-4588-y

**Published:** 2018-03-27

**Authors:** Mohammad Nur Alam, Lihua Zhang, Li Yang, Md. Rabiul Islam, Yang Liu, Hong Luo, Pingfang Yang, Qingfeng Wang, Zhulong Chan

**Affiliations:** 1 Key Laboratory of Aquatic Botany and Watershed Ecology, Wuhan Botanical Garden/Sino-Africa Joint Research Center, Chinese Academy of Sciences, Wuhan, Hubei 430074 China; 2 University of Chinese Academy of Sciences, Beijing, 100039 China; 3 Agronomy Division, Wheat Research Centre, Bangladesh Agricultural Research Institute, Joydebpur, Gazipur, 1701 Bangladesh; 4 Institute for Advanced Studies and College of Life Science, Wuhan University, Wuhan, Hubei 430072 China; 5 Institute of Poultry and Veterinarian, Hubei Academy of Agricultural Science, Wuhan, Hubei 430209 China; 6 Department of Genetics and Biochemistry, Clemson University, Clemson, USA; 7 Key Laboratory of Horticultural Plant Biology, Ministry of Education, College of Horticulture and Forestry Sciences, Huazhong Agricultural University, Wuhan, Hubei 430070 China; 8 Key Laboratory for Quality Control of Characteristic Fruits and Vegetables of Hubei Province, College of Life Science and Technology, Hubei Engineering University, Xiaogan, Hubei 432000 China

**Keywords:** 24-epibrassinolide, Antioxidant, Gene expression, Heat stress, Melatonin, Tall fescue, Transcriptomic analysis

## Abstract

**Background:**

Tall fescue is a widely used cool season turfgrass and relatively sensitive to high temperature. Chemical compounds like melatonin (MT) and 24-epibrassinolide (EBL) have been reported to improve plant heat stress tolerance effectively.

**Results:**

In this study, we reported that MT and EBL pretreated tall fescue seedlings showed decreased reactive oxygen species (ROS), electrolyte leakage (EL) and malondialdehide (MDA), but increased chlorophyll (Chl), total protein and antioxidant enzyme activities under heat stress condition, resulting in improved plant growth. Transcriptomic profiling analysis showed that 4311 and 8395 unigenes were significantly changed after 2 h and 12 h of heat treatments, respectively. Among them, genes involved in heat stress responses, DNA, RNA and protein degradation, redox, energy metabolisms, and hormone metabolism pathways were highly induced after heat stress. Genes including *FaHSFA3*, *FaAWPM* and *FaCYTC2* were significantly upregulated by both MT and EBL treatments, indicating that these genes might function as the putative target genes of MT and EBL.

**Conclusions:**

These findings indicated that heat stress caused extensively transcriptomic reprogramming of tall fescue and exogenous application of MT and EBL effectively improved thermotolerance in tall fescue.

**Electronic supplementary material:**

The online version of this article (10.1186/s12864-018-4588-y) contains supplementary material, which is available to authorized users.

## Background

Heat stress has become the major limiting factor for inhibition of plant growth and development and is causing severe reduction of crop yield worldwide [1]. In response to heat stress, various molecular pathways and relevant physiological processes were modulated in plants, resulting in increase of misfolding proteins which were bound to HSP70/90 and released HSFA1s to activate downstream heat stress responsive genes [2–5]. During plant heat stress response, a series of metabolic alterations occur, including overproduction of reactive oxygen species (ROS) and reactive nitrogen species (RNS), lipid peroxidation producing the end product like malondialdehide (MDA), photoinhibition, protein denaturation and degradation, and accumulation of compatible solutes [4–7].

Melatonin (N-acetyl-5-methoxytryptamine) was discovered by McCord and Allen [8] in bovine pineal gland and found to act as a neurohormone contributing to many physiological events in animals [9–11]. Melatonin was considered exclusively as an animal hormone till a couple of decades ago when two independent groups simultaneous had discovered melatonin in edible plants [12, 13]. Melatonin existed among the plant species from the lowest ng kg^-1^ to maximum mg kg^-1^ as dry weight [14]. Evidences showed that melatonin had functioned as a ubiquitous, amphiphilic and pleiotropic signaling molecule to modulate numerous cellular, physiological and molecular pathways in plant and animal kingdom [7, 9, 15–20]. Recent studies revealed that melatonin played the vital roles in lateral root formation, germination control, plant growth and biotic and abiotic stress responses [21–28]. It was also noted that melatonin played the key roles during leaf senescence and cell death processes in plants through regulation of genes *IAA17*, *SEN* and *SAG* [20, 24]. Brassinosteroids (BRs) were involved in numerous physiological and biochemical processes including leaf senescence, promotion of cell expansion and elongation, signal transduction, as well as adaptation to a variety of environmental stresses [29–37]. 24-epibrassinolide (EBL) and 28-homobrassinolide (HBL) are commercially available BRs. Exogenous application of EBL stimulated plant tolerance to chilling, salt, heat stress, heavy metals and oxidative stresses [38–48].

Tall fescue (*Festuca arundinacea* Schreb.), a perennial cool-season turfgrass, is one of the most important and intensively studied grass species globally [49]. Heat stress induced ROS accumulation and increased activities of antioxidant enzymes in various tall fescue accessions [4, 50]. Jiang and Huang observed that gene encoding cytosolic-heat shock protein (*HSC70*) in two tall fescue cultivars had been induced after drought and ABA treatments [51]. Moreover, heat and drought stresses simultaneously deduced the gene expression of *psbB* and *psbC*, but induced the expression of *psbA*. Two genes encoding heat shock protein (*HSP*) in tall fescue displayed higher transcript abundance after heat treatment and basal transcript level during the recovery stage [5]. An A2-type *FaHsfA2c* gene was induced by heat stress and overexpression of *FaHsfA2c* in tall fescue increased plant heat tolerance through modulation of photosynthesis and heat stress responsive genes [52]. RNA sequencing analysis identified 12,974 unigenes as differentially expressed genes in two tall fescue genotypes after long term heat stress treatment [53]. Those results provided us the valuable information to dissect the heat stress responsive mechanisms of tall fescue. However, the effects of short term heat stress on tall fescue at early development stages were elusive.

In this study, we investigated the effects of MT and EBL pretreatments on heat stress tolerance in tall fescue, and how MT and EBL affected tall fescue at physiological and transcriptomic levels. We observed that heat stress caused extensively transcriptomic reprogramming in tall fescue and application of MT and EBL on tall fescue significantly changed redox and hormone related pathways.

## Methods

### Plant material and growth conditions

Tall fescue (*Festuca arundinacea* Schreb.) variety Fire Phoenix ΙΙ was provided by Beijing Clover Group. Seeds were surface-sterilized with 5. 25% (*w*/*v*) sodium hypochlorite (NaClO), and rinsed 4 times with sterile water. After stratification at 4 °C for 4 days, the seeds were sown in MS medium plates (11. 5 cm × 11. 5 cm × 1. 5 cm) containing 1% (w/v) sucrose [54]. The plates were then kept at the growth room supplemented with 200 μmol/m^2^/s fluorescent light under the photoperiod of 16/ 8 h (light/dark), 23 °C temperature, and 65% relative humidity.

### Heat treatments and experimental design

Melatonin and 24-epibrassinolide (Sigma-Aldrich, MO, USA) were dissolved into 99.97% ethanol (w/v), then 50 mM and 1 mM stock solutions were prepared for MT and EBL, respectively. Eight days old seedlings were transplanted into the transparent plastic pots (height: 13 cm, top diameter: 9 cm, bottom diameter: 7 cm) containing MS medium supplemented with indicated amount of MT, EBL or water (Control). After 2 days cultivation, all seedlings were imposed in the growth chamber with 38 °C and 42 °C temperature for 2, 6, 12, and h. After indicated time points, the shoots of seedlings were immediately collected and stored at − 80 °C refrigerator for the physiological and biochemical analyses.

### Measurement of fresh weight and plant height of tall fescue

After heat treatment at 38 °C, all plants were transferred to room temperature for recovery. Shoot fresh weight and plant height were measured after 2 days recovery.

### Quantification of ROS, antioxidant enzymes and protein content

After heating treatment for 6 and 12 h, seedling shoot (about 0.2 g) was collected and frozen using liquid nitrogen immediately. All shoot materials were homogenized and ground with sterile pestle and mortar. Then 1. 6 mL 0.1 M sodium phosphate buffer (pH 7. 4) was added and centrifuged the tubes at 12,000 *g* at 4 °C for 15 min. The supernatant was transferred to a fresh 1. 5 ml tubes for the quantification of antioxidant enzymes, ROS and protein content.

Hydrogen peroxide (H_2_O_2_) content was measured according to the method of titanium sulfate as described by Hu et al. [55]. H_2_O_2_ content was calculated based on the standard curve and expressed as μmol g^-1^ fresh weight. Superoxide anion radical (O_2_^−^) was quantified using the Plant O_2_•^−^ ELISA Kit (10–40–488, Bejing Dingguo, Beijing, China) as previously described [27, 28]. The superoxide anion radical was calculated according to the manufacturer’s protocol and expressed as U/mg tissue.

The total protein content was measured as described by Bradford [56] using bovine serum albumin (BSA) as the standard.

The activities of catalase (CAT, EC 1. 11. 1. 6), peroxidase (POD, EC 1. 11. 1. 7) and superoxide dismutase (SOD, EC 1. 15. 1. 1)were assayed using CAT Assay Kit (A007–1, Nanjing Jiancheng, Nanjing, China), Plant POD Assay Kit (A084–3, Nanjing Jiancheng, Nanjing city, China) and Total SOD Assay Kit (S0102, Nanjing Jiancheng, Nanjing, China), respectively, as described by Shi et al. [27, 28].

### Measurement of MDA and electrolyte leakage

MDA and electrolyte leakage (EL) are the marker/indicator of cell membrane stability. The MDA content was extracted using thiobarbituric acid (TBA) regent, and quantified via determining the absorbance of the supernatant at 450 nm, 532 nm and 600 nm as previously described [28]. EL was assayed using a conductivity meter (Leici-DDS-307A, Shanghai, China) as previously described [28]. The relative EL was then calculated using the following formula: EL (%) = (C_i_/C_max_) × 100.

### Measurement of chlorophyll

Chlorophyll (Chl) content was measured according to the method as described by Frank et al. with slight modification [57]. Briefly, 100 mg fresh leaves were collected and ground with sterile pestle and mortar. The samples were transferred into 4 mL tubes, and then 2.0 mL 96% ethanol was added. After mixing well, the tubes were stratified at 4 °C for 2 days. The samples were centrifuged at 12000 *g* at 4 °C for 1 min, and then OD value of supernatant was assayed with spectrophotometer (Tecan M200 Pro, Mannedorf, Switzerland) at 665 nm and 649 nm wavelengths. Chlorophyll content (a & b) was calculated using the following formulas where A is the Absorbance [58]: Chl (a) = 13.95 × A665–6.88 × A645; Chl (b) = 24.96 × A649–7. 32 × A665.

### RNA sequencing analysis

The leaves of control and heat treated seedlings were collected for RNA isolation. RNA integrity and purity were checked with the Bioanalyzer 2100 system (Agilent Technologies, CA, USA) and the NanoPhotometer® spectrophotometer (IMPLEN, CA, USA), respectively. RNA sequencing analysis was conducted by Novogene Corporation (Beijing, China). Briefly, NEBNext® Ultra™ RNA Library Prep Kit for Illumina® (NEB, USA) was used to generate sequencing libraries with 3 μg RNA according to manufacturer’s protocol. The libraries were then sequenced on an Illumina Hiseq platform after cluster generation and 125 bp/150 bp paired-end reads were generated. Low quality reads, ploy-N from raw data and reads containing adapter were removed to generate clean reads. Transcriptome assembly was performed based on the left.fq and right.fq files using Trinity [59]. Gene function annotation was accomplished based on the databases including Nr, Nt, Pfam, KOG/COG, Swiss-Prot, KO and GO. Gene expression level changes were estimated by RSEM [60]. Differential gene expression analysis of heat stress versus control condition was performed using the DESeq R package (1. 18.0). To minimize the false discovery rate, the *p*-values were adjusted using the Benjamini and Hochberg’s approach. Genes with a fold change ≥ 2 and an adjusted p-value ≤ 0.05were assigned as differentially expressed. Two biological replicates were used for each sample. The clean data were submitted to the Gene Expression Omnibus (GEO) database with accession number of GSE101699.

### Gene ontology (GO) term and pathway enrichment and cluster analyses

Genes with *P*-value ≤ 0.05 and fold change ≥ 2 were used for GO term and pathway enrichment analyses through the Classification SuperViewer Tool (http://bar.utoronto.ca/ntools/cgi-bin/ntools_classification_superviewer.cgi) [61]. The annotated TAIR IDs of each tall fescue gene were loaded. MapMan (http://mapman.gabipd.org/home) [62] and and GO (ftp://ftp.arabidopsis.org/home/tair/Ontologies/Gene_Ontology) were used as classification sources for pathway and GO term enrichment analyses, respectively. The normalized frequency (NF) of each functional category was calculated as following: NF = sample frequency of each category in each sample/background frequency of each category in Arabidopsis genome. For hierarchical cluster analysis, the data sets of specific genes were analyzed with the CLUSTER program (http://bonsai.hgc.jp/~mdehoon/software/cluster/software.htm) using an uncentered matrix and complete linkage method [63]. The resulting tree figures were displayed using Java Treeview (http://jtreeview.sourceforge.net/) as described by Chan et al. [64].

### Quantitative real-time PCR (qRT-PCR)

Total RNA was extracted from plant shoots using Trizol reagent (Invitrogen, Carlsbad, CA, USA) and treated with RNase-free DNase (Promega, Madison, WI, USA). Five μg RNA (Conc. 500 ng/μL) DNA-free total RNA was reverse transcribed into first-strand cDNA with reverse transcriptase (TOYOBO, Ohtsu, Japan). qRT-PCR was conducted with CFX96 Real Time System (Bio-Rad, Richmond, CA, USA) with SYBR-green fluorescence. The thermal cycle program was as following: 95 °C for 5 min, 45 cycles of 95 °C for 10s and 60 °C for 30s; 95 °C for 5 min, 65 °C for 5 s, 95 °C for 1 min. Detailed fold change of each gene was calculated using the 2^−ΔΔCT^ method. Gene-specific primers for qPCR were listed in the Additional file 1: Table S1. Tall fescue *Alpha Tubulin* (Accession No. GT051159) was used as internal control.

### Statistical analysis

The whole experiments were repeated three times in this study. The mean (±SD) was the average of three biological replicates. Letters on the bar indicate significant difference at *P* ≤ 0.05 level (Duncan test) among different treatments compared to untreated control. The statistical analysis was performed using the software SPSS 16.0 followed by One-Way ANOVA.

## Results

### Effects of exogenous MT and EBL on plant growth under heat stress

**Fig. 1:**
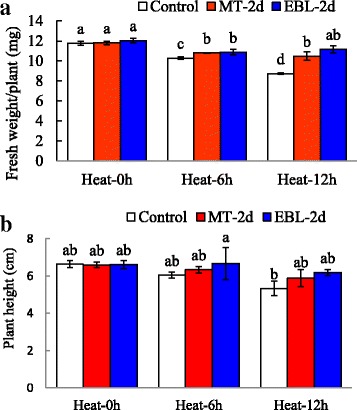
Effects of exogenous melatonin and 24-epibrassinolide on growth of tall fescue seedlings under heat stress condition. **a** Shoot fresh weight; **b** Shoot height. Eight days old seedlings on control MS plates were transplanted into MS medium containing 20 μM melatonin (MT)or 0. 1 μM 24-epibrassinolide (EBL) for 2 d, and then heat stress was applied for indicated time. Tall fescue seedlings were moved to room temperature for 2 d recovery, and then plant weight and shoot height were measured. Data were means ±SE of three biological replications. Bars with different letters indicate significant difference at *P*≤0.05 (Duncan test)

As a cool season turfgrass, tall fescue is relatively susceptible to high temperature. At 6 and 12 h after 42 °C heat treatments, only 51 and 25% plants were survival, respectively. The growth of tall fescue was severely inhibited even after very short term of heat treatment at 42 °C as evidenced by significantly decreased plant height and fresh weight after 3 h treatment (Additional file 2: Figure S1). In this study, we then selected mild high temperature at 38 °C for further experiment. Based on preliminary experiment, 20 μM MT and 0. 1 μM EBL treated tall fescue plants showed relatively lower electrolyte leakage (EL), and these concentrations were selected for the final experiment (Additional file 3: Figure S2). Heat treatment for 12 h significantly reduced plant fresh weight and slightly declined plant height in tall fescue (Fig. 1). Under heat stress condition, MT and EBL pretreated seedlings exhibited higher fresh weight than that in non-treated control (Fig. 1a, b). MT and EBL pretreatment did not affect seedling growth under non-stressed condition and plant height after heat treatment. These results indicated that MT and EBL pretreatments were effective to improve heat stress tolerance of tall fescue.

### Effects of MT and EBL on EL, chlorophyll content and total protein content under heat stress

**Fig. 2:**
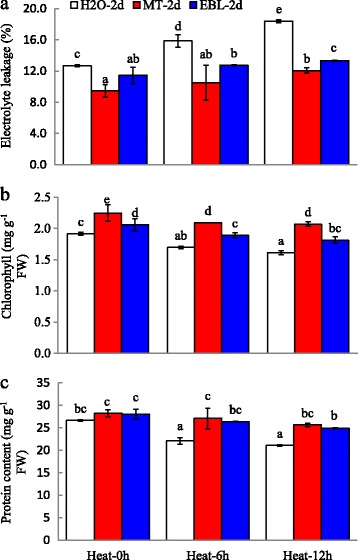
Effects of exogenous melatonin and 24-epibrassinolide on EL, chlorophyll and total protein contents of tall fescue seedlings under heat stress condition. **a**: electrolyte leakage; **b**: chlorophyll content; **c**: total protein content. Eight days old seedlings on control MS plates were transplanted into MS medium containing 20 μM melatonin (MT) or 0. 1 μM 24-epibrassinolide (EBL) for 2 d, and then heat stress was applied for indicated time. For EL and chlorophyll determination the seedlings were moved to room temperature for 2 d recovery, but plant materials was collected immediately after heat stress for protein analysis. Data were means ±SE of three biological replications. Bars with different letters indicate significant difference at *P*≤0.05 (Duncan test)

EL is the efficient indicator of cell membrane stability. Heat treatment caused significantly increased EL in all seedlings. However, MT and EBL pretreatments significantly reduced EL under heat stress condition (Fig. 2a). Heat stress treatment decreased chlorophyll and protein contents in tall fescue seedlings, indicating that heat stress largely affected plant photosynthesis and protein metabolism pathways (Fig. 2b, c). MT and EBL pre-treated plants showed significantly higher chlorophyll content and total protein content under both control and heat stress conditions when compared with controls (Fig. 2b, c). These results indicated that exogenous MT and EBL might compensate the damages caused by heat stress in tall fescue.

### Effects of MT and EBL on ROS and cell membrane stability under heat stress

**Fig. 3:**
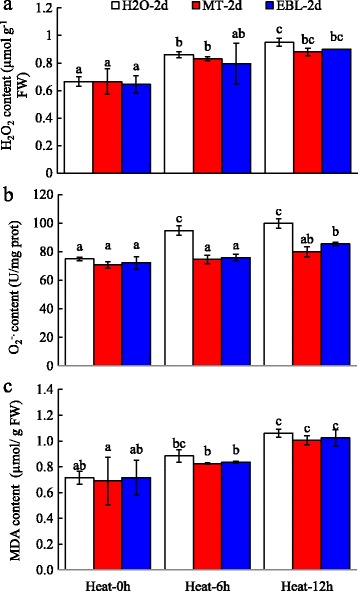
Effects of exogenous melatonin and 24-epibrassinolide on ROS content and cell membrane stability of tall fescue under heat stress condition. **a**: H_2_O_2_ content; **b**: Superoxide anion radical content; **c**. MDA content. Eight days old seedlings on control MS plates were transferred to MS medium containing 20 μM melatonin (MT)or 0. 1 μM 24-epibrassinolide (EBL) for 2 days. Then heat stress was applied for indicated time, and plant shoots were collected immediately. Data were means ±SE of three biological replications. Bars with different letters indicate significant difference at *P*≤0.05 (Duncan test)

Hydrogen peroxide (H_2_O_2_) and superoxide anion radical (O_2_^**−**^) create oxidative stress in organisms which cause bases damage of any components, as well as strand breaks in DNA and RNA. To evaluate the effects of MT and 24-EBL on ROS metabolisms in tall fescue under heat stress, the contents of H_2_O_2_ and O_2_^**−**^ were measured. As shown in Fig. 3, H_2_O_2_ and O_2_^**−**^ production significantly increased after heat treatment when compared to control samples grown at 23 °C (Fig. 3a, b). Moreover, MT and EBL pre-treated seedlings exhibited lower and O_2_^**−**^ content than non-pretreated control plants when grown under heat stress condition. These results indicated that MT and EBL could ameliorate O_2_^**−**^ generation after heat stress treatment.

Abiotic stress caused lipid peroxidation. The end products of lipid peroxidation are reactive aldehydes, such as malondialdehide (MDA) and 4-hydroxynonenal (HNE). Heat treatment for 12 h significantly increased MDA content in tall fescue. Exogenous MT and EBL pre-treatments slightly decreased MDA production, but no significant differences were observed after MT and EBL pretreatments (Fig. 3c). These results indicated that MT and EBL alleviated cell membrane damage caused by heat stress.

### Effects of MT and EBL on antioxidant enzyme activities under heat stress

**Fig. 4:**
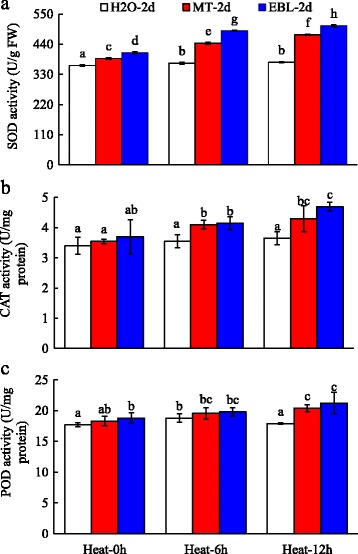
Effects of exogenous melatonin and 24-epibrassinolide on the activities of antioxidant enzymes of tall fescue under heat stress. **a**: SOD; **b**: CAT; **c**: POD. Eight days old seedlings on control MS plates were transferred to MS medium containing 20 μM melatonin (MT)or 0. 1 μM 24-epibrassinolide (EBL) for 2 days, and then heat stress was applied for indicated time, and plant materials were collected immediately. Data were means ±SE of three biological replications. Bars with different letters indicate significant difference at *P*≤0.05 (Duncan test)

Under heat stress condition, activity of SOD showed significant increase at 2 and 12 h, and POD at 2 h after treatment. Heat stress did not significantly affect CAT activity (Fig. 4a–c). Pre-treatments with MT and EBL significantly increased SOD activity at 0, 2, 12, and h, CAT activity at 2 and 12 h, and POD activity at 12 h after heat treatment (Fig. 4a–c). These results indicated that MT and EBL pre-treatments increased antioxidant enzyme activities which might be responsible for decreased ROS content (Fig. 4).

### General transcriptomic profiling of tall fescue to heat stress

**Fig. 5:**
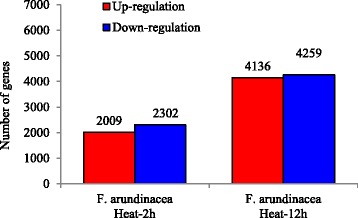
Number of unigenes was significantly changed by heat stress in tall fescue. Unigenes with putative annotation were analyzed and full list was presented in Additional file 5: Table S3. Ten days old seedlings were treated with heat stress for indicated time

**Fig. 6:**
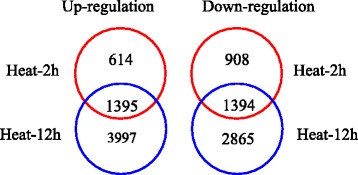
Overlapping analysis of transcripts in tall fescue after short term (2 h) and long term (12 h) heat stress treatment. Unigenes with putative annotation were analyzed and the full list was presented in Additional file 5: Table S3. Ten days old seedlings were treated with heat stress for the above indicated times

To dissect the detailed mechanisms of tall fescue in response to heat stress, RNA sequencing analysis was performed to identify heat stress modulated genes in tall fescue. Short term (2 h) and long term (12 h) heat stress treatments were used in this study. In total 134,261,972 raw reads were generated. After removing low quality reads, reads containing adapter and ploy-N from raw data, 129,334,586 clean reads and 16. 16 G clean bases were generated for the six samples used in the study (Additional file 4: Table S2). In parallel experiment, other 10.03 G clean bases were generated and we used total 26. 19 G clean bases data for transcriptome assembly. After gene differential expression analysis, 9878 unigenes were regulated after heat treatment (Additional file 5: Table S3). Transcriptomic data showed that 2009 and 2302 unigenes were up- and down-regulated after short term (2 h) heat stress treatment, respectively, whereas 4136 and 4259 unigenes were up- and down-regulated after long term (12 h) heat stress treatment, respectively (Fig. 5). These results indicated that long term heat treatment caused relatively more extensive transcriptomic changes than short term heat treatment. Moreover, 1395 unigenes were up-regulated by both short and long term heat treatments, while 1394 unigenes were down-regulated by both heat treatments (Fig. 6). These genes modulated by both short and long term heat treatments might play the key roles in responses of tall fescue to heat stress.

### GO term and pathway enrichment analyses

**Fig. 7:**
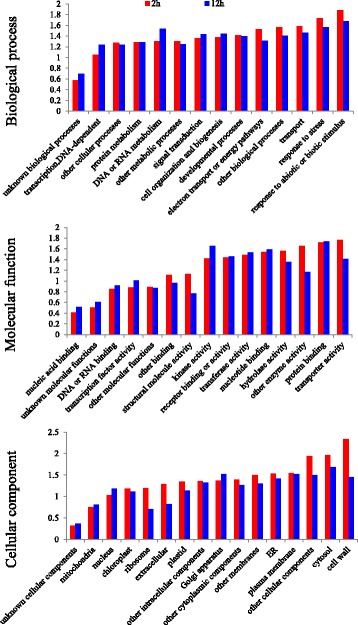
GO term enrichment analysis of differentially expressed transcripts in tall fescue after heat treatment. Differentially expressed transcripts with putative annotation to Arabidopsis were used for GO term analysis. The list of genes were submitted to The Bio-Analytic Resource for Plant Biology (http://bar.utoronto.ca/welcome.htm) with the analysis tool “Classification SuperViewer” and “GO” was selected as classification source

The enriched biological process GO terms include response to abiotic or biotic stimuli, response to stress, transport, and DNA or RNA metabolism, etc. (Fig. 7). The enriched molecular function GO terms include transporter activity, protein binding, kinase activity, and hydrolase activity, etc. (Fig. 7). The enriched cellular component GO terms include cell wall, cytosol, other cellular component, plasma membrane and ER, etc. (Fig. 7). Pathway enrichment analysis showed that 6 MapMAN pathways, including major carbohydrate (CHO) metabolism, glycolysis, N-metabolism, minor CHO metabolism, amino acid metabolism, and Co-factor and vitamin metabolism were enriched after both short term and long term heat treatments. Short term heat treatment specifically affected pathways like nucleotide metabolism, glycolysis, oxidative phosphorylation (OPP), fermentation, photosynthesis (PS), and tetrapyrrole synthesis etc. while long term heat treatment changed the pathways of polyamine metabolism, metal handling and redox etc. (Table 1).

**Table 1:**
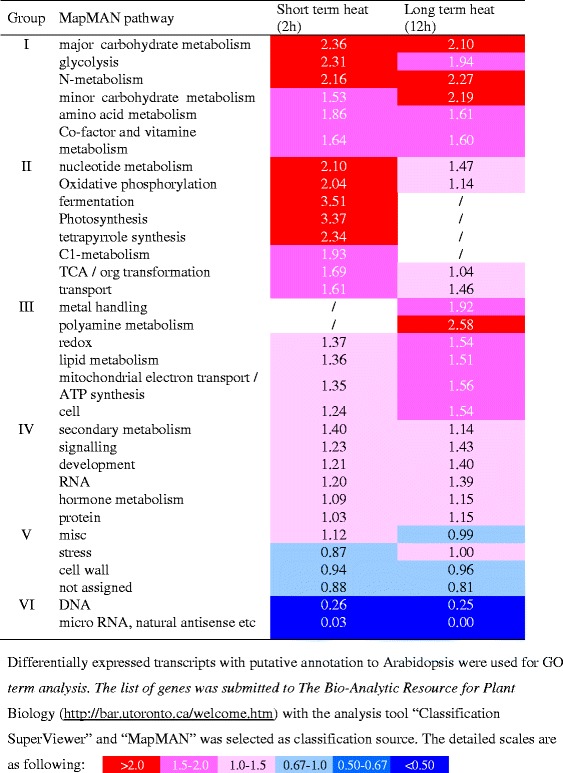
MapMan pathway analysis of differentially expressed transcripts in tall fescue after heat treatment

### Cluster analysis and functions of heat stress responsive genes

**Fig. 8:**
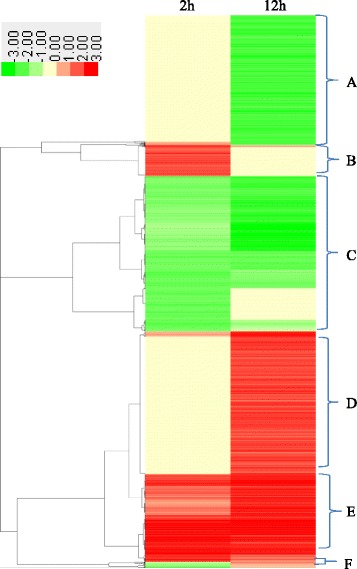
Cluster analysis of transcripts in tall fescue after heat treatment. In total 9878 transcripts changed in tall fescue after 2 or 12 h heat treatment was used for cluster analysis. The lists of genes were analyzed using CLUSTER 3.0 and the resulting tree figure was shown by Java Treeview. The original data were presented in Additional file 6: Table S4

**Fig. 9:**
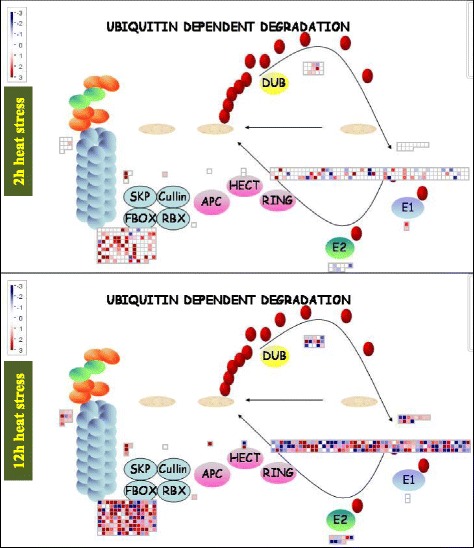
Heat stress modulated ubiquitin dependent protein degradation pathway in tall fescue. The pathway was generated using MapMAN software (Version 3. 6.0RC1). Totally 344 genes were involved in ubiquitin dependent protein degradation pathway (Additional file 7: Table S5). Each square means a gene. Red color means up-regulation and blue color means down-regulation

Cluster analysis showed that the part of unigenes were specifically regulated by short term or long term heat treatment (Fig. 8a, b, d, f; Additional file 6: Table S4), but other unigenes were co-regulated by both short term and long term heat treatments (Fig. 8c, e). Therefore, short term and long term heat stress had the specific and common effects on transcriptome of tall fescue. Moreover, detailed metabolic pathway analysis showed that ubiquitin dependent protein degradation pathway had been activated after heat stress treatment (Fig. 9). In total 344 differentially expressed genes were involved in proteasome directed protein degradation pathway (Additional file 7: Table S5). Among them, 76 and 31 genes were up- and down-regulated by 2 h heat, respectively, while 197 and 125 genes showed the increase- and decrease trend after 12 h heat treatment, respectively (Additional file 7: Table S5). These data indicated that heat stress had accelerated protein degradation in tall fescue.

Functions of highly induced unigenes by both short term and long term heat stress were further analyzed. Totally 89 genes exhibited at least 32-fold changes (log2≥ 5) after heat treatments (Additional file 8: Table S6). Among them, HSF, HSP and HSC were heat stress responsive genes in plants. HSP chaperones are responsible primarily with protein folding and/or assembly. Up-regulation of these genes helps plant to buffer stress induced damage. Several genes encoding ribonuclease, DNA polymerase, protease, hydrolases, RING/U-box and F-box showed significant increase after short term and long term heat stress, indicating plant suffered from heat induced damage and degradation pathway was activated (Additional file 8: Table S6). Additionally, genes involved in photosynthesis and energy metabolism pathways including those encoding ATPase, cytochrome C, transferase and dehydrogenase were also highly induced. Other genes encoding kinase, splicing factor and transporter were significantly up-regulated under heat condition (Additional file 8: Table S6).

### Changes of hormone pathway related genes

Based on transcriptomic analysis, we observed that many hormone related genes, including BR, MT, auxin (IAA), and abscisic acid (ABA) showed significant changes after heat stress treatment (Additional file 9*:* Table S7). Genes involved in BR, MT and IAA biosynthesis and signaling pathways were mainly downregulated, while the majority of genes functioned in ABA pathway were up-regulated (Additional file 9: Table S7).

### Effects of MT and EBL on the expression of heat stress induced genes

**Fig. 10:**
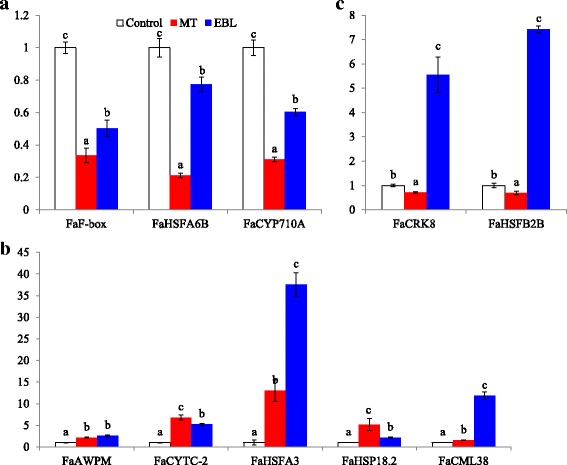
qPCR analysis of heat stress induced genes in tall fescue. Eight days old seedlings grown on control MS plate were transferred to MS medium containing 20 μM melatonin (MT) or 0. 1 μM 24-epibrassinolide for 2 days, and then heat stress (38 °C) was applied for indicated time. Shoots of seedlings were collected immediately for RNA extraction. Notably, 20 genes highly induced by heat stress were selected for qPCR analysis and ten of them showed significant changes after MT or EBL treatments. The detailed information of selected genes and primers used for qPCR were listed in Additional file 1: Table S1. A: genes downregulated by both MT and EBL; B: genes upregulated by both MT and EBL; C: genes upregulated only by EBL, d, day; h, hour. Data were means ±SE of three biological replications. Bars with different letters indicate significant difference at *P*≤0.05 (Duncan test)

As mentioned above, 89 genes showed at least 32-fold changes (log2≥ 5) after heat treatments in tall fescue (Additional file 8*:* Table S6)*.* From these heat stress highly induced genes, twenty genes were selected for qRT-PCR analysis. Among them, ten genes showed significant expression changes in seedlings after MT or EBL pretreatment (Fig. 10). Three genes including *FaF-box*, *FaHSFA6B* and *FaCYP710A* were significantly down-regulated by both MT and EBL pretreatments (Fig. 10a). Five genes including *FaAWPM*, *FaCYTC*-2, *FaHSFA3*, *FaSHP18.* 2 and *FaCML38* showed significant up-regulation in MT and EBL pretreated tall fescue seedlings (Fig. 10b). Two genes including *FaCRK8* and *FaHSFB2B* were significantly up-regulated after EBL treatment, but showed a slight down-regulation by MT treatment (Fig. 10c). The results indicated that several genes might be the putative targets of MT or EBL during tall fescue heat stress response (Fig. 10).

## Discussion

Under heat stress condition, plants develop various strategies to buffer the damages caused by heat, including physiological, biochemical and transcriptomic levels. Tall fescue is a wide used cool season grass and relatively sensitive to heat stress. We hypothesized that heat stress responsive genes would be induced by a relatively short term heat treatment. In this study, the results showed that growth and physiological parameters of tall fescue plants were mainly inhibited by heat treatment, while pre-treatment with MT and EBL partially impaired inhibitory effect of heat stress (Figs. 1 and 2). The similar results were observed by Shi et al. and Antoniou1 et al. who found that exogenous application of melatonin improved heat tolerance of Arabidopsis and drought tolerance of alfalfa, respectively [17, 65]. Moreover, exogenous application of melatonin in bermudagrass grown under oxidative stress resulted in improved growth as evidenced by higher fresh weight and plant height [28]. Zhang et al. observed increased shoot and root fresh mass and chlorophyll content in EBL pretreated melon plants than those of control [66]. The positive correlation of EBL with plant growth under heat stress condition was also proven by Zhang et al. [22] and Kumar et al [67]. These results indicated that molecules like MT and EBL might be effective to improve the thermotolerance in plants.

Generally, heat stress stimulates the formation of RNS (like NO) and ROS, such as OH^−^, H_2_O_2_, and O_2_^**−**^, resulting in increased electrolyte leakage and the lipid peroxidation, and the activities of antioxidant enzymes are enhanced following heat treatment. Among them, superoxide radical (O_2_^**−**^) is dismutated by SOD into H_2_O_2_ and is further scavenged by CAT and peroxides (such as POD) through converting into H_2_O. EBL treatment helped to ameliorate abiotic stresses by regulating the activities of antioxidant enzymes and oxidants [22, 46, 67, 68]. Exogenous application of MT was proved to mitigate oxidative damages caused by abiotic stresses to maintain ROS homeostasis [17, 28, 69]. In this study, the activities of CAT, SOD and POD increased (Fig. 4), but the content of oxidants decreased in tall fescue after exogenous application of MT and EBL which improved heat stress tolerance in tall fescue by enhancing plant growth (Fig. 1). These results were consistent with the findings of other reporters who concluded that MT and EBL pretreatments alleviated oxidative stress in plants [7, 17, 35, 39, 42]. Mazorra et al. found that thermotolerance was independent for endogenous BRs content, but heat stress-mediated oxidative stress was depended on BRs [70]. Therefore, the decrease of oxidants and elevation of reductant/antioxidant enzymes production in MT and EBL pretreated seedlings were involved in the increase of plant heat tolerance.

On the basis of transcriptomic analysis, we identified several heat stress responsive genes in tall fescue like U-box and *F-box proteins*, *HSP*, *HSF* and chaperone DnaJ-domain superfamily proteins etc. Chaperones proteins assist the covalent folding or unfolding and the assembly or disassembly of other macromolecular structures, especially protein folding [69, 70]. As shown in Additional file 8: Table S6, *HSP20*-like chaperones protein (c152923_g1, c152923_g5), and DnaJ-domain superfamily protein (c142517_g1) of tall fescue showed significantly higher expression after short and long term heat stresses. Moreover, several heat shock transcription factors were induced by heat stress, including *HSFA* and *HSFB* gene family (Additional file 7: Table S5 and Fig. 10). *HSF*s were the transcriptional regulators that specifically bind to DNA sequence 5‘-AGAAnnTTCT-3‘known as heat shock promoter elements (HSE), and then regulate expression of downstream heat shock protein genes [2]. One gene encoding *HSFB2a* (c139552_g1) induced by heat more than 50-fold was also modulated by MT (Additional file 8: Table S6), *FaHSFA3* was significantly upregulated, while *FaHSFB2B* was slightly downregulated by MT in this study. The same trend was observed in warm season turfgrass *Cynodon dactylon* after MT treatment based on RNA seq analysis [27]. The results indicated that these genes were possibly involved in improved heat stress responses by MT (Fig. 10).

Interestingly, heat stress increased the expression of genes encoding ribonuclease, RNA-directed DNA polymerase, protease, hydroxylase. Genes involved in ubiquitination related pathways were also up-regulated after heat treatment (Additional file 7: Table S5). All these genes play key roles during degradation pathway of DNA, RNA, and protein. To date, not much attention was paid to dissect the roles of RNase and DNA polymerase during plant abiotic stress response. How protein ubiquitination modulated heat response in tall fescue is also worthy to be further explored. Additionally, genes encoding protein kinases were upregulated after 2 and 12 h of heat treatments (Additional file 7: Table S5). Plant receptor-like protein kinase (RLK) was proved to be involved in abiotic stress responses [71]. Modulation of cysteine-rich receptor-like protein kinase affected plant ABA sensitivity and improved stress tolerance in Arabidopsis [72, 73].

Abiotic stresses conferred severe damage on the photosynthetic machinery of plants [74]. In the study, genes involved in energy metabolism (glycolysis, ATP biosynthesis, photosynthesis) were highly induced after heat stress, including these encoding mitochondrial ATPase, ferredoxin, cytochrome C, and phosphoribosyltransferase (Fig. 10; Additional file 7: Table S5). Heat stress caused the reduction of chlorophyll content in tall fescue (Fig. 2b), thus leading to induce photosynthetic pathways. Genes encoding proline and nitrate transporters were upretulated up to 235- and 90-fold, respectively (Additional file 7: Table S5). The up-regulation of these genes implied that plants had tried to maintain the steady photosynthesis and minimize the damages caused by high temperature (Figs. 2 and 3).

Hormones like IAA, MT and BRs stimulate plant growth at appropriate low concentrations [19, 32, 40]. In tall fescue, heat stress treatment resulted in downregulation of genes involved in biosynthesis and signaling pathways of IAA, MT and BRs (Additional file 9*:* Table S7). Genes involved in brassinosteroid pathway, including *DWF4* (key enzyme during biosynthesis pathway), *BRI1* (BR receptor), BR-responsive *RING* were downgregulated by heat treatment. Two genes (*ASMT* and *SNAT*) involved in MT biosynthesis showed significant decrease upon heat treatment. Therefore, heat stress inhibited, while MT and EBL improved growth and thermotolerance of tall fescue (Fig. 1). Our data are consistent with the results that MT and EBL application increased thermotolerance in other plant species [20, 27, 39]. Additionally, ABA plays the vital roles in plant stress responses. *NCED3*, a key enzyme in the biosynthesis of ABA, was upregulated 20-fold in tall fescue after heat stress treatment (Additional file 9*:* Table S7). Another ABA biosynthesis gene (*ABA2*), The majority of PP2C genes, *SnRK2.* 6*/OST1* and downstream ABA responsive genes like *ABF2* and *ABF3* showed significant increase upon heat treatment (Additional file 8*:* Table S7). These genes were essential for ABA signaling and plant stress response [75, 76]. Increased ABA content and upregulation of ABA responsive genes activated the downstream transcriptional factors and ABA pathway. Our data is in line with the results in Arabidopsis that ABA pathway genes were activated after abiotic stress treatment [77]. These results indicated that hormones might function as the upstream regulator in tall fescue during heat stress responses.

## Conclusion

**Fig. 11:**
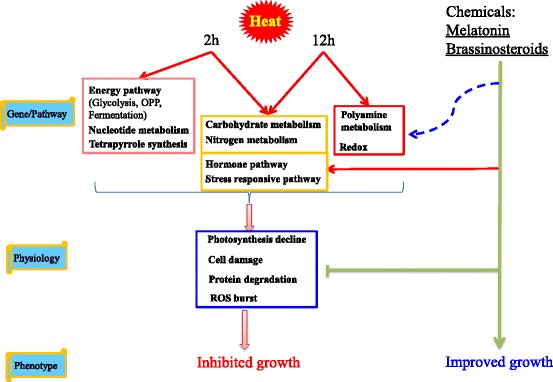
A model depicts inhibitory effect of heat stress on growth of tall fescue and improved growth by exogenous melatonin (MT) and 24-epibrassinolide (EBL). Heat stress affected nucleotide metabolism, carbohydrate metabolism, nitrogen metabolism, polyamine metabolism, redox, hormone pathway, energy pathway, and regulated expression of stress responsive genes, resulting in inhibited photosynthesis, increased cell damage, degradation of protein and other macromolecules, and increased ROS content. Growth of tall fescue was then inhibited. However, exogenous application of chemicals like MT and EBL modulated expression of stress responsive genes, as well as genes involved in hormone biosynthesis and signaling pathways, and then alleviated inhibitory effects of heat stress, leading to improved growth of tall fescue plants

In this study, heat stress extensively changed transcriptome of tall fescue. Degradation pathways of DNA, RNA and protein were activated by heat. After heat treatment, genes involved in energy metabolism were upregulated to minimize photosynthesis damage caused by heat. *HSF*, *HSP* and hormone related genes were modulated shortly after heat treatment, indicating self-adjustment of plant in response to heat stress. Short term heat stress activated nucleotide metabolism and tetrapyrrole synthesis, while long term heat stress regulated polyamine metabolism and redox pathways. Moreover, exogenous application of MT and EBL improved tall fescue tolerance to heat stress as evidenced by decreased ROS level, EL, MDA content, and increased chlorophyll content, antioxidant enzyme activities. Therefore, heat stress inhibited plant growth while MT and EBL alleviated the inhibitory effect of heat (Fig. 11). These findings along with excellent results by other groups highlighted the significance and potential use of MT and EBL agents to improve plant abiotic stress tolerance.

## Additional files

Additional file 1: **Table S1**. List of primers used for real-time PCR. (XLSX 11 kb)

Additional file 2: **Figure S1**: Effect of high temperature at 42 °C on growth of tall fescue seedlings. (PPTX 90 kb)

Additional file 3: **Figure S2**: Concentration screening of exogenous melatonin and 2, 4-epibrassinolide on tall fescue seedlings under heat stress condition. (PPTX 157 kb)

Additional file 4: **Table S2**. Summary of transcriptome samples. (XLSX 8 kb)

Additional file 5: **Table S3**. List of gene changed by heat stress in tall fescue. (XLSX 1578 kb)

Additional file 6: **Table S4**. List of genes used for cluster analysis. (XLSX 563 kb)

Additional file 7: **Table S5**. List of 344 genes involved in ubiquitin dependent protein degradation pathway. (XLSX 67 kb)

Additional file 8: **Table S6**. List of highly induced genes in tall fescue by heat stress. (XLSX 18 kb)

Additional file 9: **Table S7**. List of genes involved in hormone pathways. (XLSX 28 kb)

## Abbreviations

CAT: Catalase
EBL: 24-epibrassinolide
EL: Electrolyte leakage
GO: Gene ontology
HSF: Heat shock factor
HSP: Heat shock protein
MDA: Malondialdehide
MT: Melatonin
OD: Optic density
POD: Peroxidase
RNS: Reactive nitrogen species
ROS: Reactive oxygen species
SOD: Superoxide dismutase
