# Supplementary figures and images for: Transcriptomic profiling of tall fescue in response to heat stress and improved thermotolerance by melatonin and 24-epibrassinolide
Source: BMC Genomics. 2018 Mar 27;19:224. doi: 10.1186/s12864-018-4588-y (PMC5870388; doi:10.1186/s12864-018-4588-y)

## Slide 1
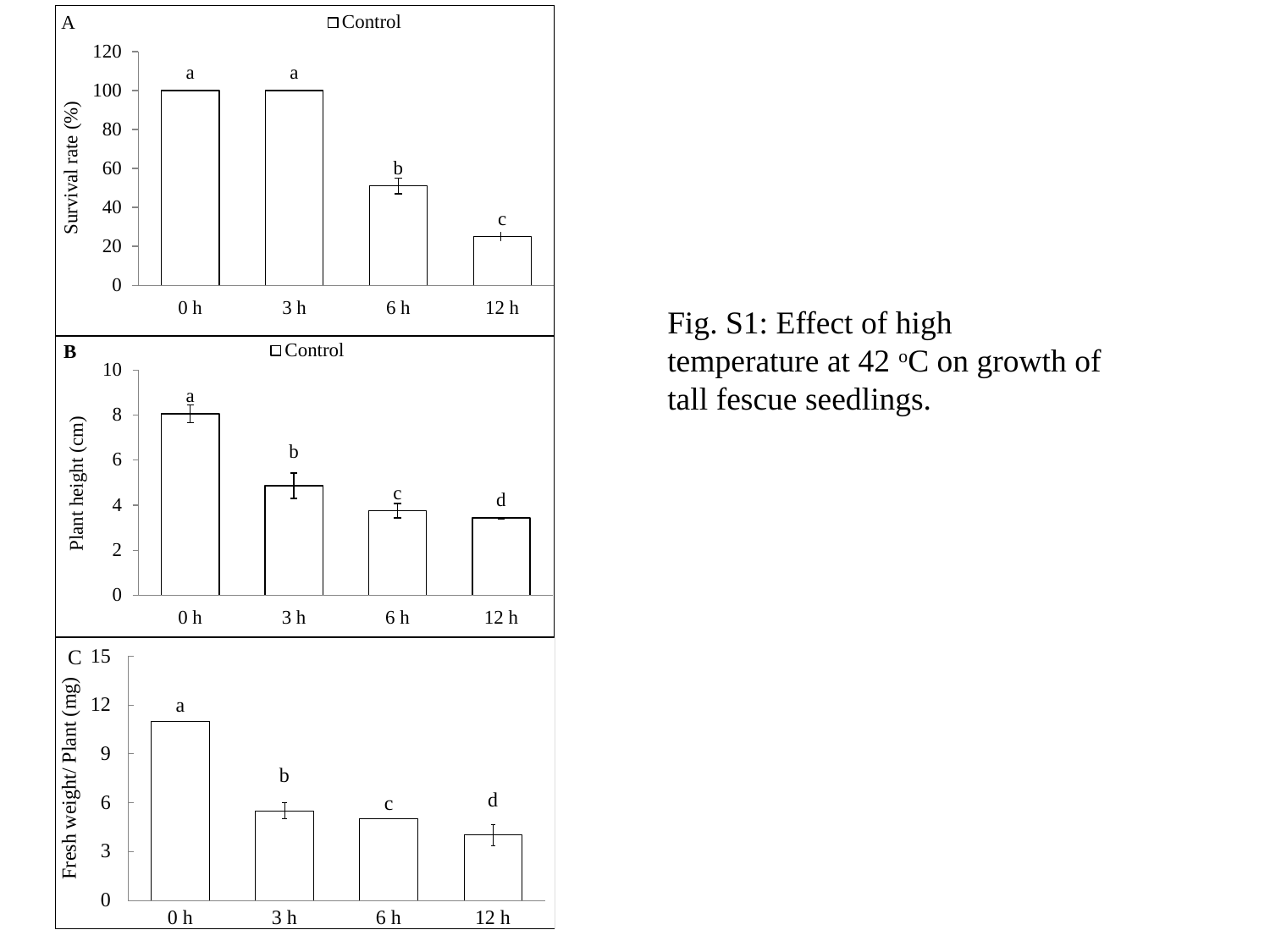

Fig. S1: Effect of high temperature at 42 oC on growth of tall fescue seedlings.

Supplement: Supplementary file 2 — Figure S1: Effect of high temperature at 42 °C on growth of tall fescue seedlings. (PPTX 90 kb) [file 12864_2018_4588_MOESM2_ESM.pptx]
