# Supplementary material for: Transcriptomic profiling of tall fescue in response to heat stress and improved thermotolerance by melatonin and 24-epibrassinolide
Source: BMC Genomics. 2018 Mar 27;19:224. doi: 10.1186/s12864-018-4588-y (PMC5870388; doi:10.1186/s12864-018-4588-y)
Supplement: Supplementary file 3 — Figure S2: Concentration screening of exogenous melatonin and 2, 4-epibrassinolide on tall fescue seedlings under heat stress condition. (PPTX 157 kb) [file 12864_2018_4588_MOESM3_ESM.pptx]

## Slide 1
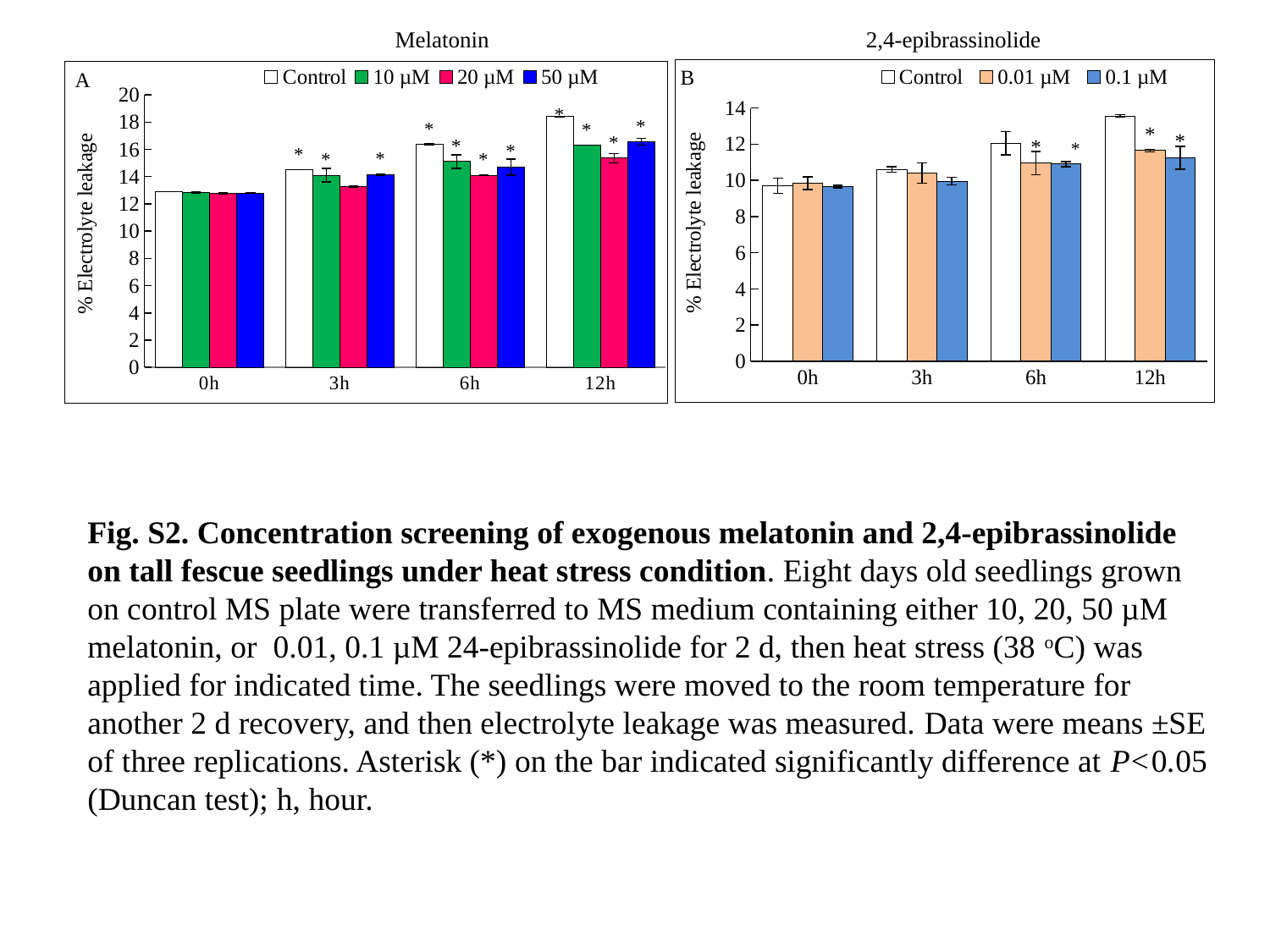

Melatonin
2,4-epibrassinolide
### Chart
| Category | Control | 0.01 µM | 0.1 µM |
|---|---|---|---|
| 0h | 9.7 | 9.85 | 9.65 |
| 3h | 10.6 | 10.4 | 9.95 |
| 6h | 12.05 | 10.95 | 10.9 |
| 12h | 13.55 | 11.65 | 11.25 |
### Chart
| Category | Control | 10 µM | 20 µM | 50 µM |
|---|---|---|---|---|
| 0h | 12.9 | 12.85 | 12.75 | 12.8 |
| 3h | 14.5 | 14.1 | 13.25 | 14.15 |
| 6h | 16.35 | 15.1 | 14.1 | 14.7 |
| 12h | 18.4 | 16.3 | 15.35 | 16.55 |Fig. S2. Concentration screening of exogenous melatonin and 2,4-epibrassinolide on tall fescue seedlings under heat stress condition. Eight days old seedlings grown on control MS plate were transferred to MS medium containing either 10, 20, 50 µM melatonin, or 0.01, 0.1 µM 24-epibrassinolide for 2 d, then heat stress (38 oC) was applied for indicated time. The seedlings were moved to the room temperature for another 2 d recovery, and then electrolyte leakage was measured. Data were means ±SE of three replications. Asterisk (*) on the bar indicated significantly difference at P˂0.05 (Duncan test); h, hour.
